# Mechanical properties of lithium slag recycled aggregate concrete subject to high temperature

**DOI:** 10.1371/journal.pone.0315133

**Published:** 2025-02-20

**Authors:** Jiongfeng Liang, Ying Yang, Caisen Wang, Ziyi Hu, Wei Li

**Affiliations:** 1 Faculty of Civil & Architecture Engineering, East China University of Technology, Nanchang, China; 2 College of Architecture and Civil Engineering, Beijing University of Technology, Beijing, China; 3 College of Civil and Architecture Engineering, Wenzhou University, Wenzhou, China; 4 Key Laboratory of Engineering and Technology for Soft Soil Foundation and Tideland Reclamation of Zhejiang Province, Wenzhou, P.R.China; Covenant University, NIGERIA

## Abstract

In attempting to enhance the mechanical properties of recycled concrete after high temperature and solve the problem of large stacking of lithium slag (LS), this paper proposes lithium slag recycled concrete (LSRAC). In this research, LS was used to replace part of the cement (*γ*_*L*_ = 10%, 20%, and 30%), recycled coarse aggregate (RCA) completely replaced the natural aggregate (*γ*_*R*_ = 100%), and the heated temperatures were 200°C, 400°C, and 600°C. This paper carried out the heating test and the strength tests. The test results indicated, for the same heating temperature, the loss of strength of LSRAC was less than that of RAC and the compressive strengths and splitting strength of LSRAC with 20% lithium slag replacement rate were improved by 33.9%, 36.5% and 34.5%, respectively. The increase in flexural strength of LSRAC with 10% lithium slag dosage reached 24.1%. The results indicate LSRAC can effectively improve the bearing capacity of structural concrete subject to high temperature. The strength retention equations of LSRAC were established by comparing the strengths of 20°C. The calculation results of the strength retention formula for post-high-temperature LSRAC matched the measured results well. Therefore, this paper provided reliable experimental basis and theoretical guidance for on-site rescue, post-disaster assessment and reinforcement of RAC used for pavement base and public facilities constructions, and the eco-friendly way for sustainable development.

## 1. Introduction

Globally, solid waste management poses a significant challenge to countries as a result of the construction boom brought about by industrial growth and rapid urbanization [1]. With the advent of environmental sustainability, the green economy and concepts such as "peak carbon" and "carbon neutrality", and the increasing number of regulations limiting greenhouse gas emissions and waste disposal, the demand for green concrete of construction is becoming more and more urgent [2]. The construction industry is moving towards green sustainability. The large quantity of construction solid waste from infrastructure creates significant opportunities for the development of green infrastructure using solid waste. Many researchers have studied this construction solid waste recycled aggregates [3–8]. Because of the attachment of RCA to the old mortar, it makes RAC have more interfacial transition zones (ITZ), which results in mechanical properties that are not as good as these of natural aggregate concrete (NAC) [8]. If the high-quality mortar is attached to RCA, RCA can be used to fully replace NAC [9]. For structural concrete, mechanical treatment is used for the old mortar wrapped around RCA to improve the bond strength with bars, which is considered as the most economical and eco-friendly method [10]. For simplifying the crushing process and improving production efficiency, large-size recycled coarse aggregate with 30% dosage reduces compressive strength by 12% [11]. Concrete with the addition of washed recycled fine aggregate has reduced strength but has good durability [12]. In view of fly ash, silica fume, ground granulated blast slag and fibers can increase the properties of NAC [5, 12–16], which makes the performance improved, even better than NAC [17–19]。

The generation of LS has also been increasing year by year, more polluting to the environment. And the annual discharge of lithium slag from optical lithium-ion batteries in China will reach about 1.25 million tons. At present, the use of LS is mainly concentrated in the production of building materials. The main reasons are the filling effect of LS [20, 21] and its pozzolanic activity [22, 23], which make microscopic pores refine and improve microstructure and ITZ [24]. Based on this property, LS was selected as a supplementary cementitious material (SCM) for concrete. The 28d compressive strength of the mortar specimens with LS replacing 40% of cement could reach 93% of that of cement specimens [23]. For recycled concrete with replaced 20% of cement by LS, the optimal mechanical properties of the specimens were obtained [24, 25]. For 50% replacement of RCA replacement rate, LS improved the strength of RAC by more than 17% [25], while other supplementary cementitious materials (SCMs) r0se by 4% [27]. This also indicated that LS has great application prospects for RAC.

Meanwhile, with urban expansion and residential concentration, the frequency of fires has gradually increased. Building fires accounted for 80% of the total fires, which greatly increased the difficulty of firefighting and aroused a great deal of attention to building fire prevention. To expand applications of RAC in actual engineering, it is crucial to acquaint the mechanical properties subject to mega temperatures. The increased temperature will affect ITZ performance between the old and new interfaces of RAC. It has been proved that the bond strength of the interface region is weakened at elevated temperatures due to the encapsulation of old mortar on the outside of RCA, which make the mechanical properties decrease [26]. To enhance the performance of RAC, it must normally be improved by SCMs [27–30] and fibers [31–33]. This is because the chemical reaction between the admixtures and cement generates supplementary cementitious materials (SCMs) that greatly improve the cement composition and concrete properties, increase the ITZ density of RAC at high temperatures, and inhibit early microcracking [24, 34]. Many researchers have found that LS is similar to SCMs mentioned above in terms of its main components, all of which have high pozzolanic activity. Theoretically, LS can improve the ability of RAC to resist elevated temperatures.

Nowadays, numerous literatures have focused on properties of LS or recycled concrete at ambient conditions, but few have analyzed mechanical properties of high-temperature LSRAC with "double waste", industrial waste and solid waste. In this paper, LS and RCA were used to replace cement and NCA, respectively. Since the properties of NAC and RAC with 20% LS replacement rate is optimal, the LS replacement rate is selected as 10%, 20%, and 30%. To consume the largest amount of RCA and also the worst performance of RAC with 100% RCA substitution rate, the rate was selected as 100%. This paper investigated the mechanical properties of LSRAC subject to high temperature by analyzing the mass loss rate and strengths and proposed the strength retention formula of LSRAC after high temperature for the application of the actual engineering fire resistance properties. LSRAC can reduce costs by at least 20% [35] and carbon emissions by approximately 18% [36]. This work will increase the range of applications for recycled concrete, especially for improving the capacity of construction engineering subject to elevated temperature [37, 38], meanwhile, this utilization can achieve the sustainable development of “double waste”.

## 2. Materials and methodology

### 2.1 Test materials

In these tests, recycled coarse aggregate (RCA) was taken from concrete crushed from building and laboratory beams and plates. The water absorption of RCA was 3.2%, the crushing index was 12.0%, and the apparent density was 2535 kg/m3. Particle gradations of RCA were listed in Table 1.

**Table 1:** Coarse aggregate particle gradation.

Size/mm		26.5	19.0	16.0	9.5	4.75
Cumulative screening margin (%)	RCA	0	11	24	79	98

**Fig 1:**
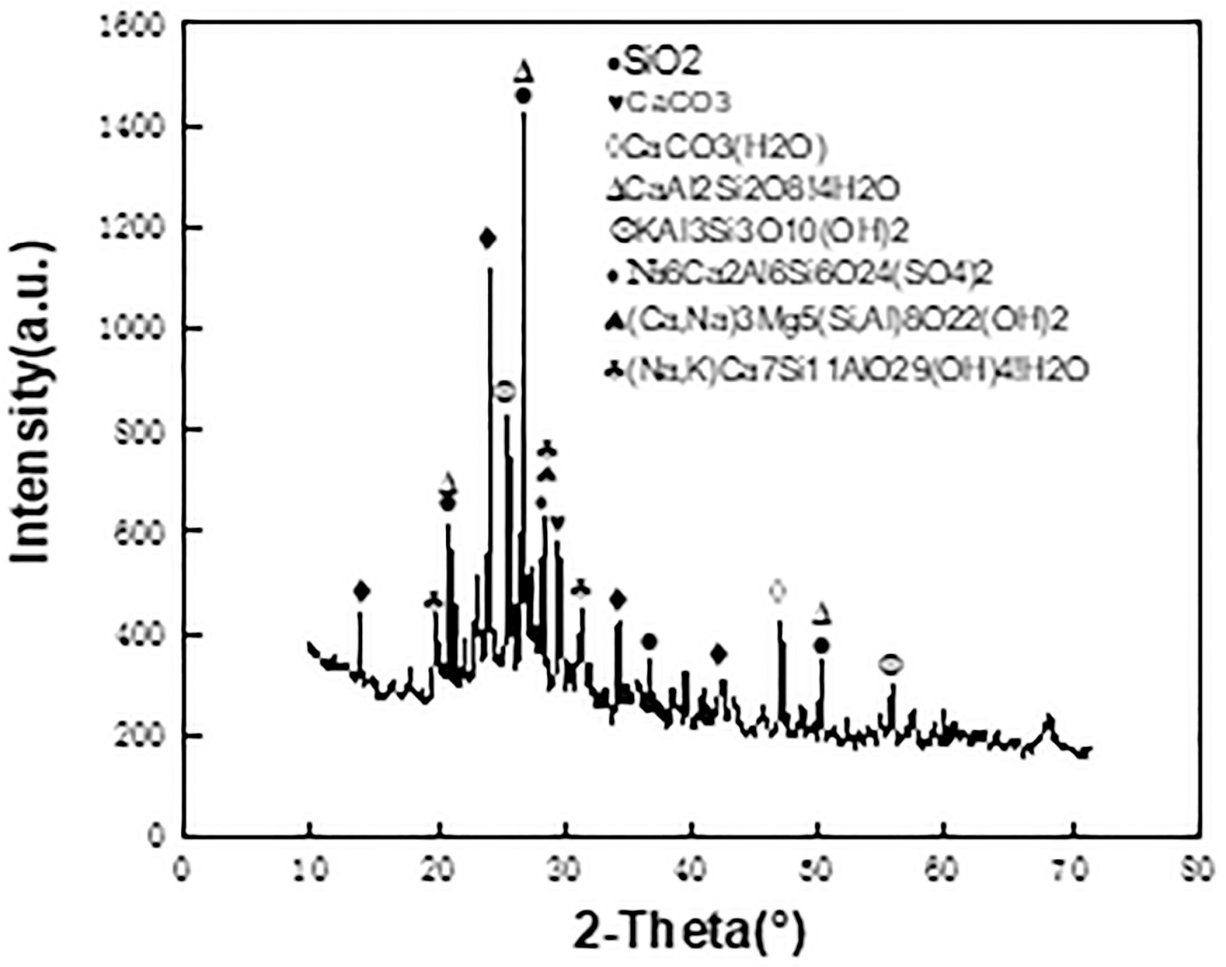
Diffraction pattern of lithium slag.

**Fig 2:**
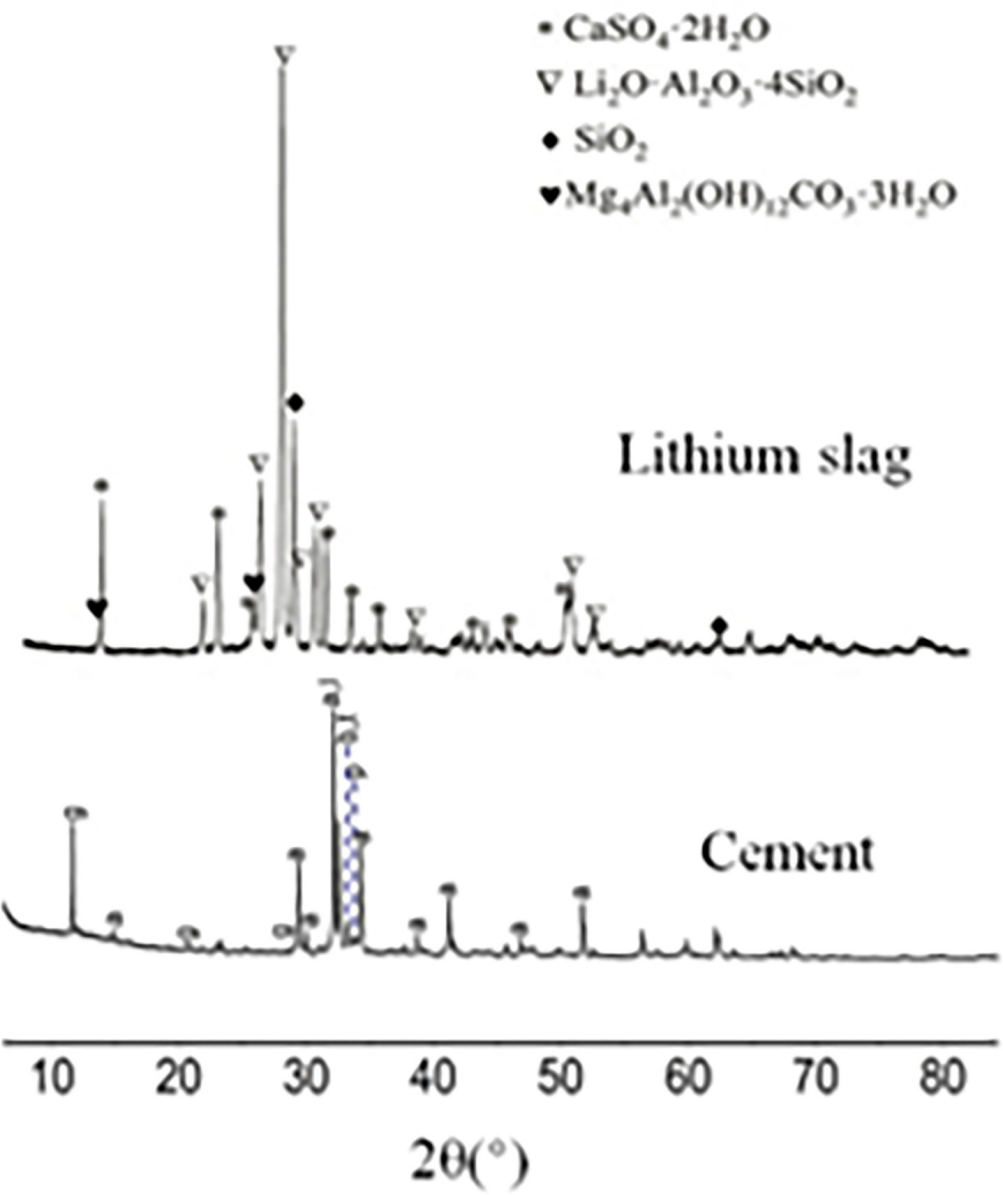
Diffraction patterns of cement and lithium slag [23].

The LS was provided by a company in Xinyu, Jiangxi Province, of which the diffraction pattern was shown in Fig 1. Fig 2 was showed the cross-reference to cement. In these tests, P.O 32.5 cement was used. Table 2 listed the principal chemical composition of cement and LS. The test water was the laboratory tap water, which met the requirements of the concrete mixture water specification.

**Table 2:** The chemical composition of cement and LS.

Material	CaO	SiO_2_	Al_2_O_3_	Fe_2_O_3_	SO_3_	MgO	Loss
Cement	60.4	18.9	5.4	4.1	2.6	1.9	6.7
LS	10.3	57.3	17.1	1.3	3.1	0.7	10.2

### 2.2 Mixture ratio

According to JGJ55–2011 [39], the concrete with a strength grade of C30 was prepared. Table 3 presented the mixture ratio of LSRAC. The concrete with various mix ratios was mixed for 6 min in an electric mixer, and then poured into a mold to make the test specimens. The specimens were unmolded after 24 hours, and then placed in a curing room with a temperature of (20 ± 2)°C and a relative humidity of more than 95% for 28 days. Compression test and split tensile test used 100mm×100mm ×100mm concrete cube specimens, Axial compressive test used the 100mm×100mm×300mm ones, flexural test used the 100mm×100mm×400mm ones.

**Table 3:** Mixture ratio.

Number	Replacement	Replacement	LS	Sand	NCA	RCA	Cement	Water	Additional water
	rate of LS	rate of RCA							
	/%	/%							
			/(kg/m^3^)	/(kg/m^3^)	/(kg/m^3^)	/(kg/m^3^)	/(kg/m^3^)	/(kg/m^3^)	/(kg/m^3^)
LR-1	0	100	0	612	0	1137	446	205	24.33
LR-2	10	100	44.6	612	0	1137	401.4	205	24.33
LR-3	20	100	89.2	612	0	1137	356.8	205	24.33
LR-4	30	100	133.8	612	0	1137	312.2	205	24.33

### 2.3 Test procedure

#### 2.3.1 Heating test

**Fig 3:**
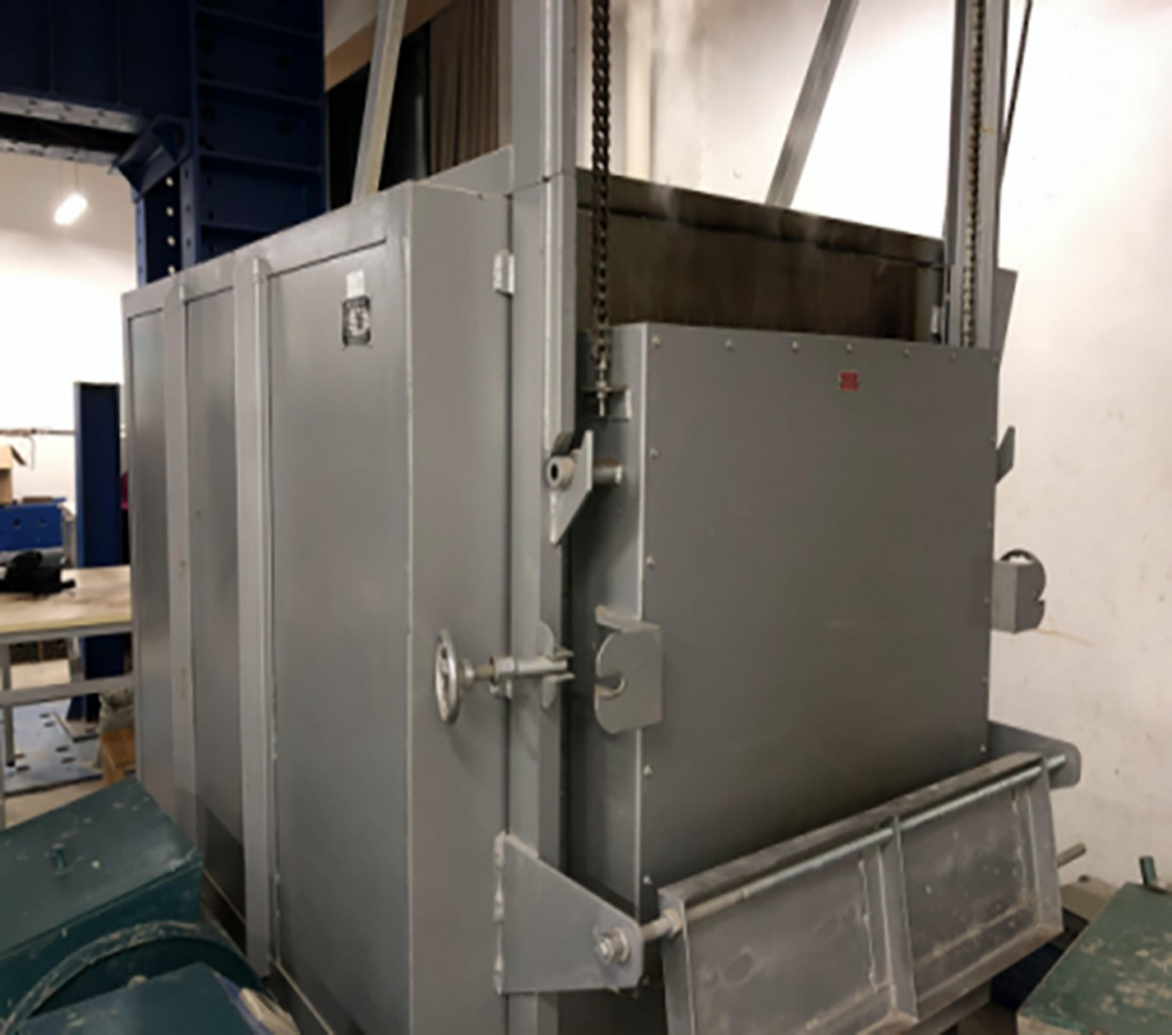
Large-size chamber resistance furnace.

**Fig 4:**
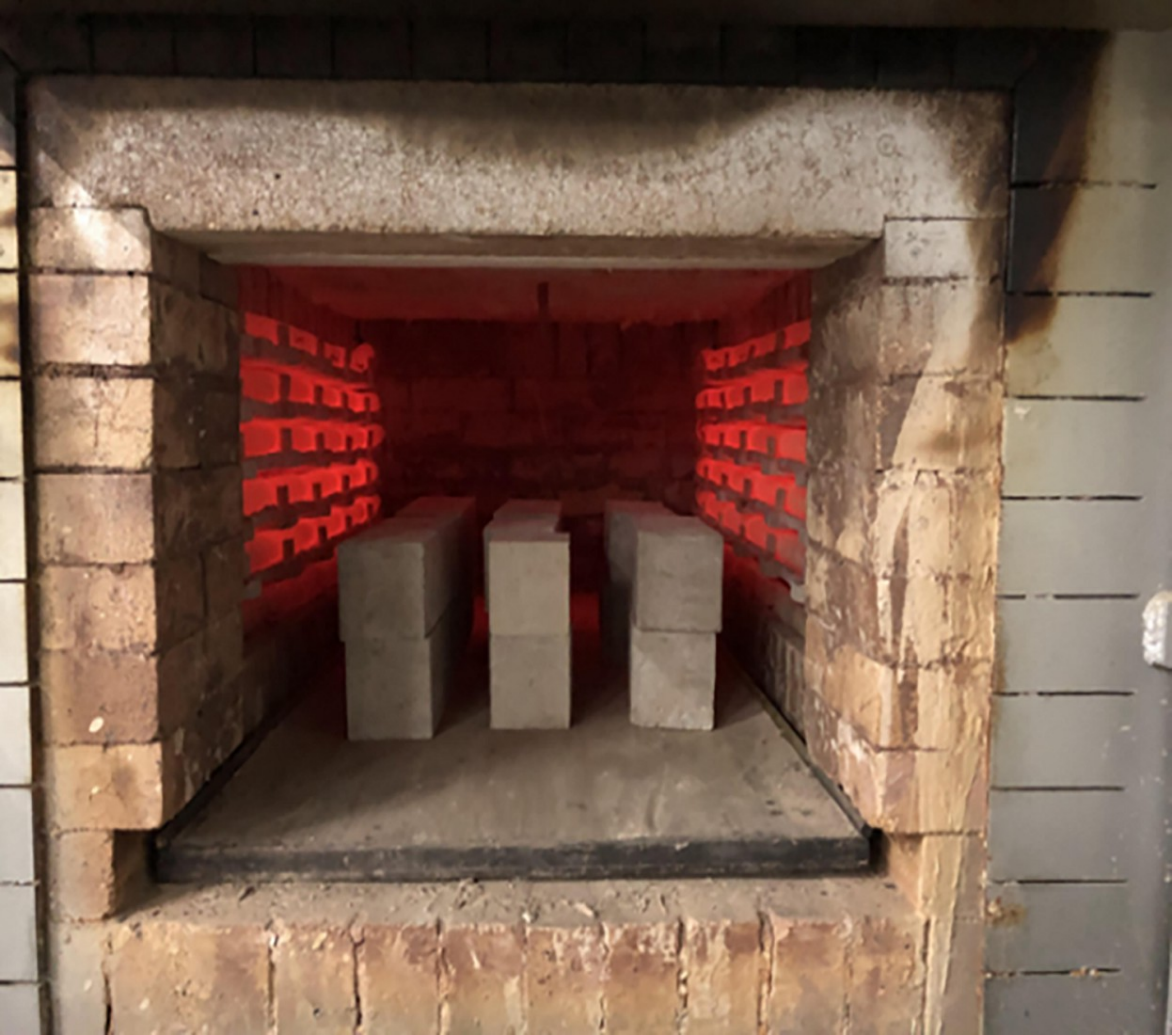
High temperature test.

To simulate the high-temperature environment of the fire, a large-size box-type resistance furnace model was used for heating, which has the highest temperature of 950°C. Due to the obvious decrease of the performance of RAC beyond 400°C [40], three heating values of 200°C, 400°C and 600°C were adopted in this paper. The specimens were numbered and weighed before the elevated temperature test and placed in the resistance furnace at equal intervals. Firstly, the resistance furnace was set to the target temperature and began to heat up according to ISO-834. When the furnace temperature reached the target value, it turned off the furnace and opened the door after keeping the constant temperature for 2 hours, took out the specimens of the cooled group and left them for 2–3 days until they were completely cooled down and then weighed. The specimens were weighted for calculating the mass loss rate at room temperature, and carried out the mechanical property test. Figs 3 and 4 showed the large-size chamber resistance furnace and high temperature test.

#### 2.3.2 Strength test

The physical and mechanical properties of LSRAC were tested according to GB/T 50080–2016 [41] and GB/T 50081–2019 [42]. After natural drying and cooling down, the specimens were tested on the pressure testing machine with a range of 3000kN. Table 4 listed the test results.

**Table 4:** The test results of LSRAC under different temperatures.

Test results	T (°C)	*γ*(%)			
		0	10	20	30
*f*_*cu*_ (MPa)	20°C	29.5	29.9	31.5	26.2
	200°C	24.3	24.5	25.3	22.9
	400°C	21.2	24.9	28.4	19.9
	600°C	13.2	14.4	15.7	13.1
*f*_*c*_ (MPa)	20°C	22.4	26.9	30.4	22
	200°C	14.4	20.1	21.5	13.6
	400°C	11.5	15.1	15.7	12.8
	600°C	7.3	10.2	10.4	9.6
*f*_*t*_ (MPa)	20°C	2.76	2.85	2.58	2.37
	200°C	2.03	2.18	2.73	2.51
	400°C	1.63	2.04	2.13	1.76
	600°C	0.81	0.98	1.16	1.14
*f*_*f*_ (MPa)	20°C	3.5	2.8	2.3	2.2
	200°C	2.9	3.6	3.0	2.6
	400°C	1.4	2.6	2.2	2.0
	600°C	0.3	0.5	0.3	0.3

## 3. Results of strength tests

### 3.1. Heating phenomenon

**Fig 5:**
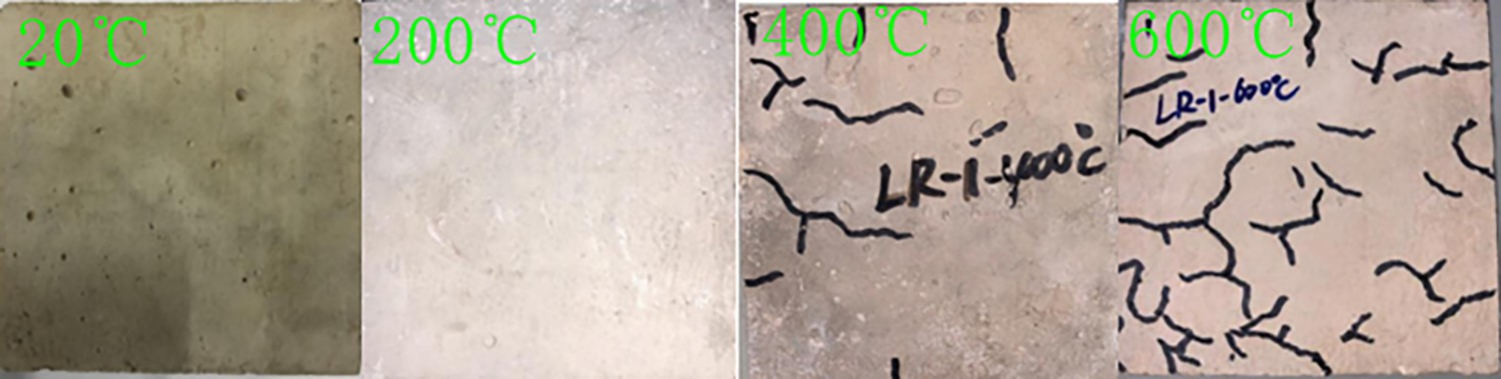
LR-1 specimens for different temperature.

**Fig 6:**
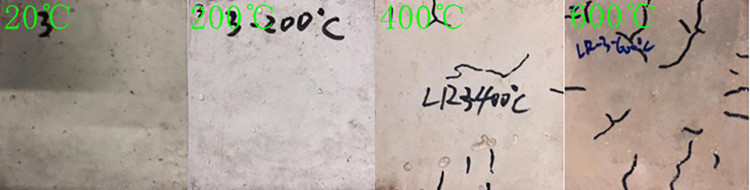
LR-3 specimens for different temperature.

After high-temperature heating of LSRAC, when the temperature was below 200°C, a small amount of white mist wafted out from the furnace wall. When the temperature reached 400°C, a large amount of water vapor appeared above the furnace wall. When the temperature arrived at 600°C, there was a popping sound. Concrete cracks, looseness, and corner dropping occurred, and micro-cracks continued to extend, eventually forming a network. When the temperature rose from 20°C to 600°C, the color of the specimen surface changed from dark gray to light gray, then to light white, and finally to light red, seen in Figs 5 and 6. The rate of mass loss of LSRAC increased with increasing heat temperature, ranging from 2.26% to 14.15%.

The high temperature caused physical and chemical reactions in LSRAC, resulting in surface color changes, cracking, and peeling of the specimens. The decomposition of Ca(OH)_2_ resulted in a grayish-white color, and the impurities inside the water reacted with water to form a scale, presenting a light red color [43]. The loss of free water and bound water during the heating process resulted in a change in weight loss and break.

### 3.2 Cubic compressive strength

The cubic compressive strength (*f*_*cu*_) for each specimen was obtained in the compressive test. Its value was the arithmetic mean for each group. The following strengths were calculated in the same way as it.

**Fig 7:**
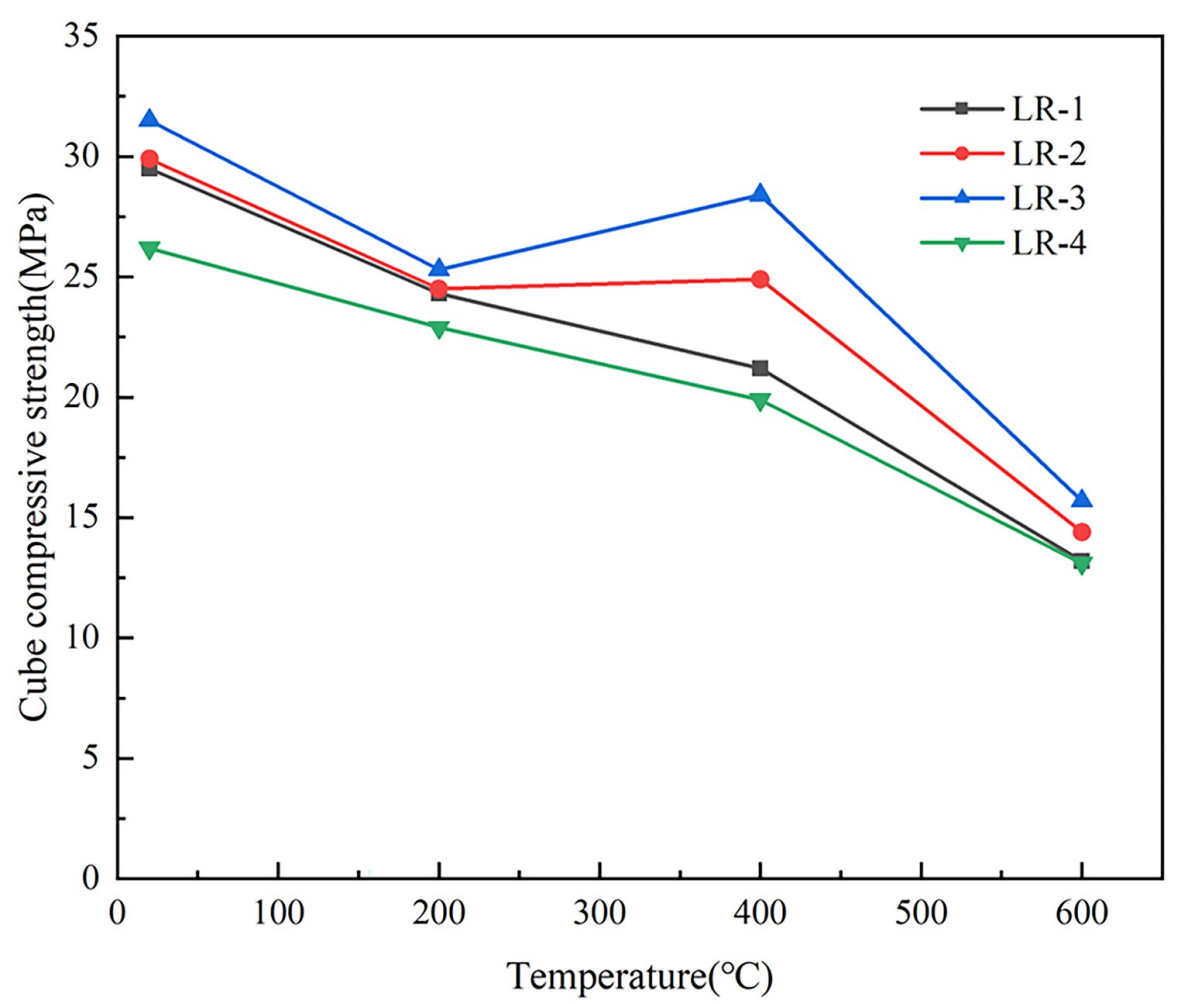
Cubic compressive strength versus temperature.

Fig 7 showed that *f*_*cu*_ of the specimens for RAC all decreased gradually with increasing heat temperature, while LSRAC specimens mixed with 10% and 20% LS all decreased firstly, then increased and then decreased with increasing heat temperature. After heating to 200°C, the retention rate of *f*_*cu*_ of the specimens decreased, and the range was 80.3–87.4%. At 400°C, the *f*_*cu*_ of the LSRAC specimens with 10% and 20% lithium slag doping increased significantly compared with that of 200°C, and the retention rate of *f*_*cu*_ was 83.3% and 90.2%, respectively. The *f*_*cu*_ retention rate of LSRAC specimens mixed with 30% lithium slag still maintained a decreasing trend, but the rate of decrease slowed down slightly, and its *f*_*cu*_ retention rate was 76.0%. From 400°C to 600°C, all specimens’ *f*_*cu*_ cut down sharply, and the retention rate of *f*_*cu*_ varied between 44.7% and 50.0%. The above analysis illustrated that when the heated temperature was more than 400°C, *f*_*cu*_ of LSRAC specimens decreased sharply, and the degree of deterioration increased with the rise of temperature; while the LSRAC in the section from 200°C to 400°C had a better ability to withstand high temperature.

**Fig 8:**
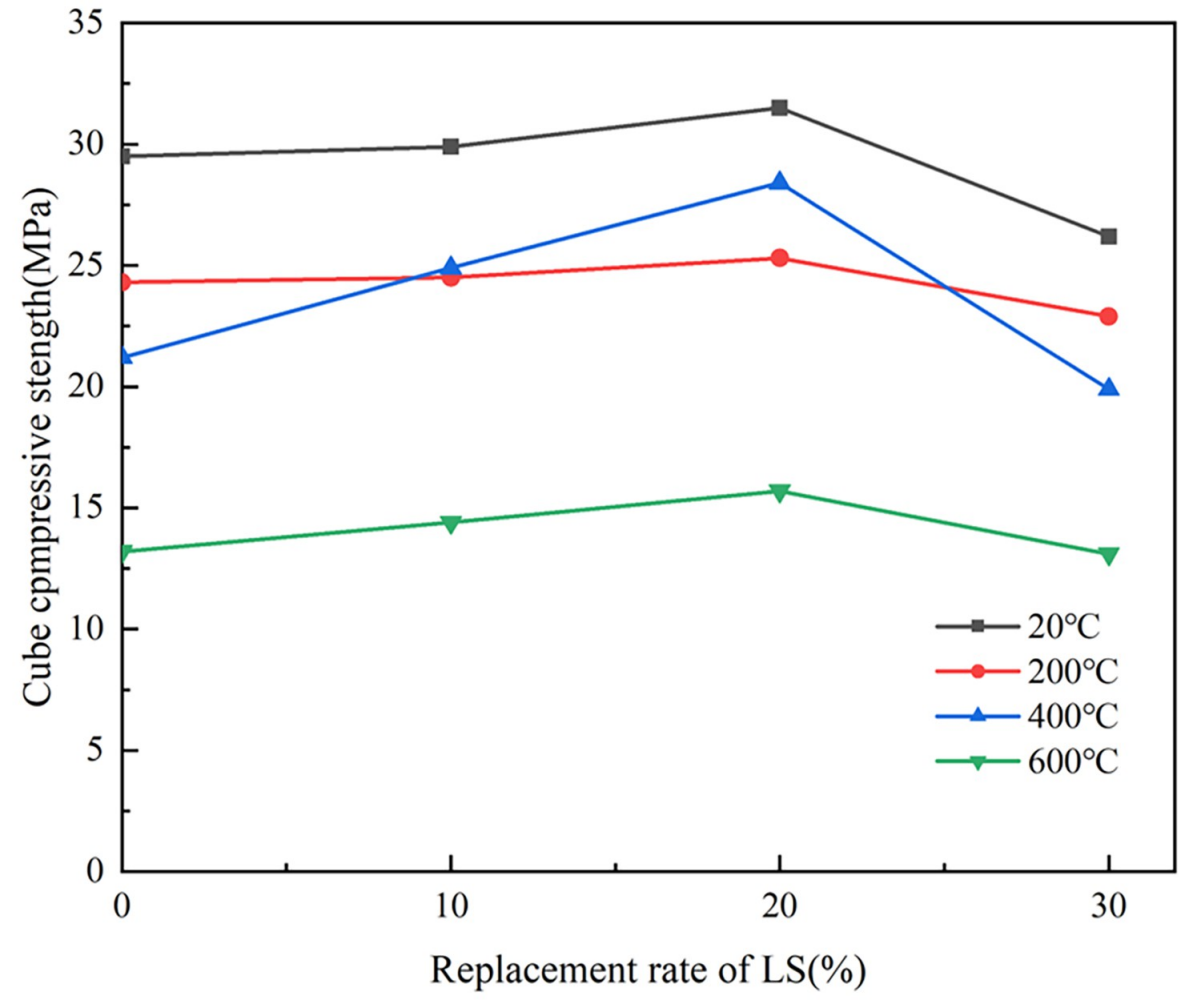
Cubic compressive strength versus replacement of LS.

Fig 8 illustrated the relationship between *f*_*cu*_ of LSRAC with 100% replacement of RCA and various replacements of LS when the temperatures were 20°C, 200°C, 400°C and 600°C. It could be presented that, *f*_*cu*_ of LSRAC firstly increased and then decreased with the lithium slag dosage for the same temperature. With 10% replacement ratio, the increase rate of *f*_*cu*_ was between 0.8% and 17.5%; when the replacement was 20%, the increase rate of strength was between 4.1% and 34.0%; when the replacement was 30%, the increase rate of strength was between -11.2% and -0.8%. From the test data, it could be concluded that the optimum lithium slag dosage was 20%, because the strength of LSRAC increased rate under different high temperatures was higher than that of the other lithium slag dosages.

The improvement of the strength of LSRAC by lithium slag was related to the reaction of CaO, SO_3_ in lithium slag with the composition and hydration products of cement to produce gypsum and calcium alumina dehydrate. The micro-expansion slowed down the self-contraction of concrete, so that the concrete was denser, and the initial cracks within the concrete became less [23, 25]. Mixed into the excessive lithium slag, there was no reaction with the hydration products of lithium slag remaining, only to play a filling role, high temperature water continued to dissipate. As a result, the internal structure of LSRAC became looser, which lowered the compressive strength. When the temperature exceeds 400 degrees, the main reason for the decline of strength was the complete disintegration of mortar due to the decomposition of the decomposition of C-H crystal and C-S-H [35].

### 3.3 Axial compressive strength

During the axial compressive test, with the load increasing, vertical cracks began to appear in the middle or lower edge of the prism. A small amount of concrete bulged out and fell off at the edges and corners of the specimens. After reaching the peak load, suddenly a "bang" noise, and the prismatic cylinder brittle damaged. The cross-section basically formed a "herringbone"-shaped diagonal crack, and the angle of destruction ranged from 60~79°.

**Fig 9:**
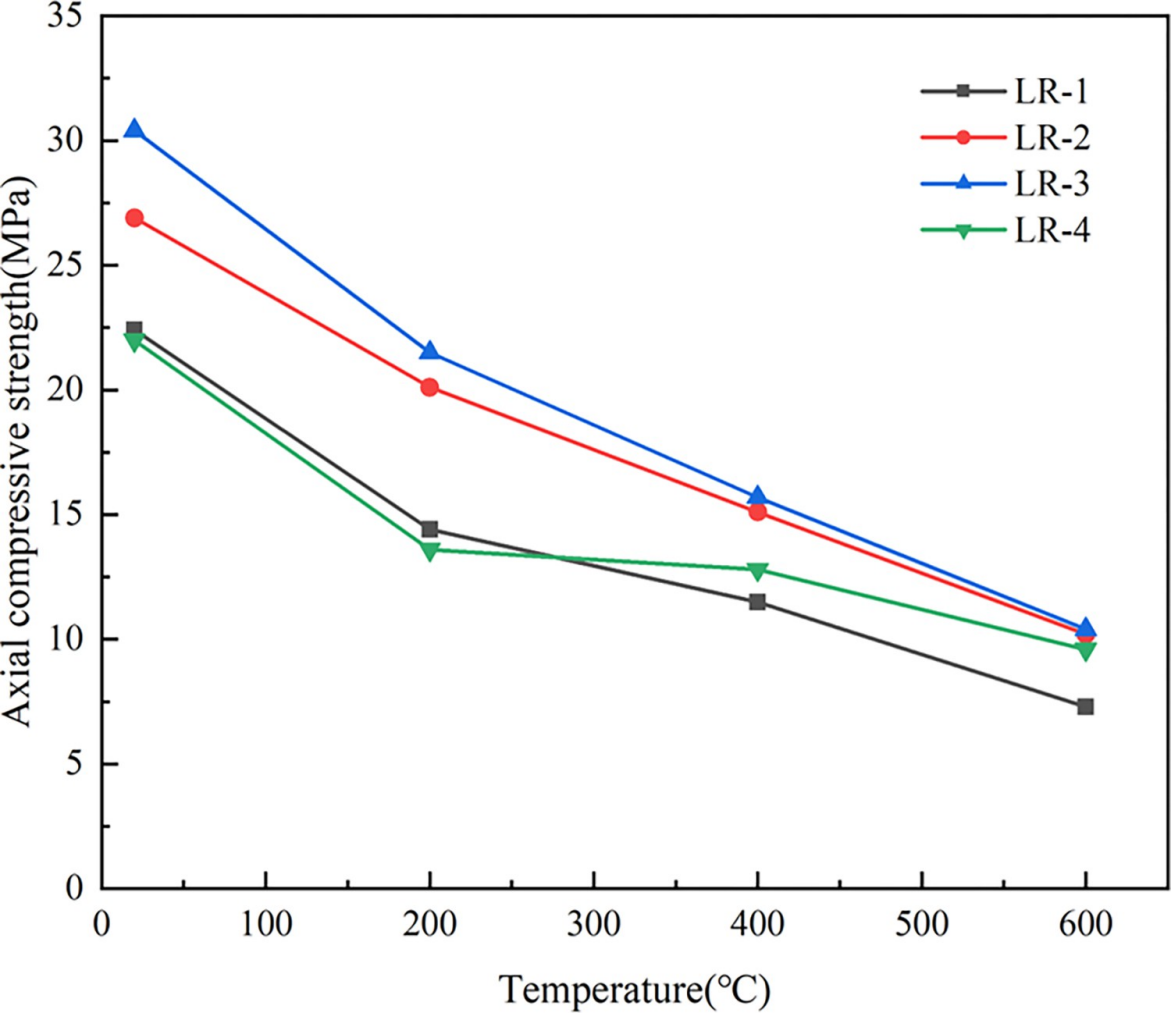
Axial compressive strength versus temperature.

**Fig 10:**
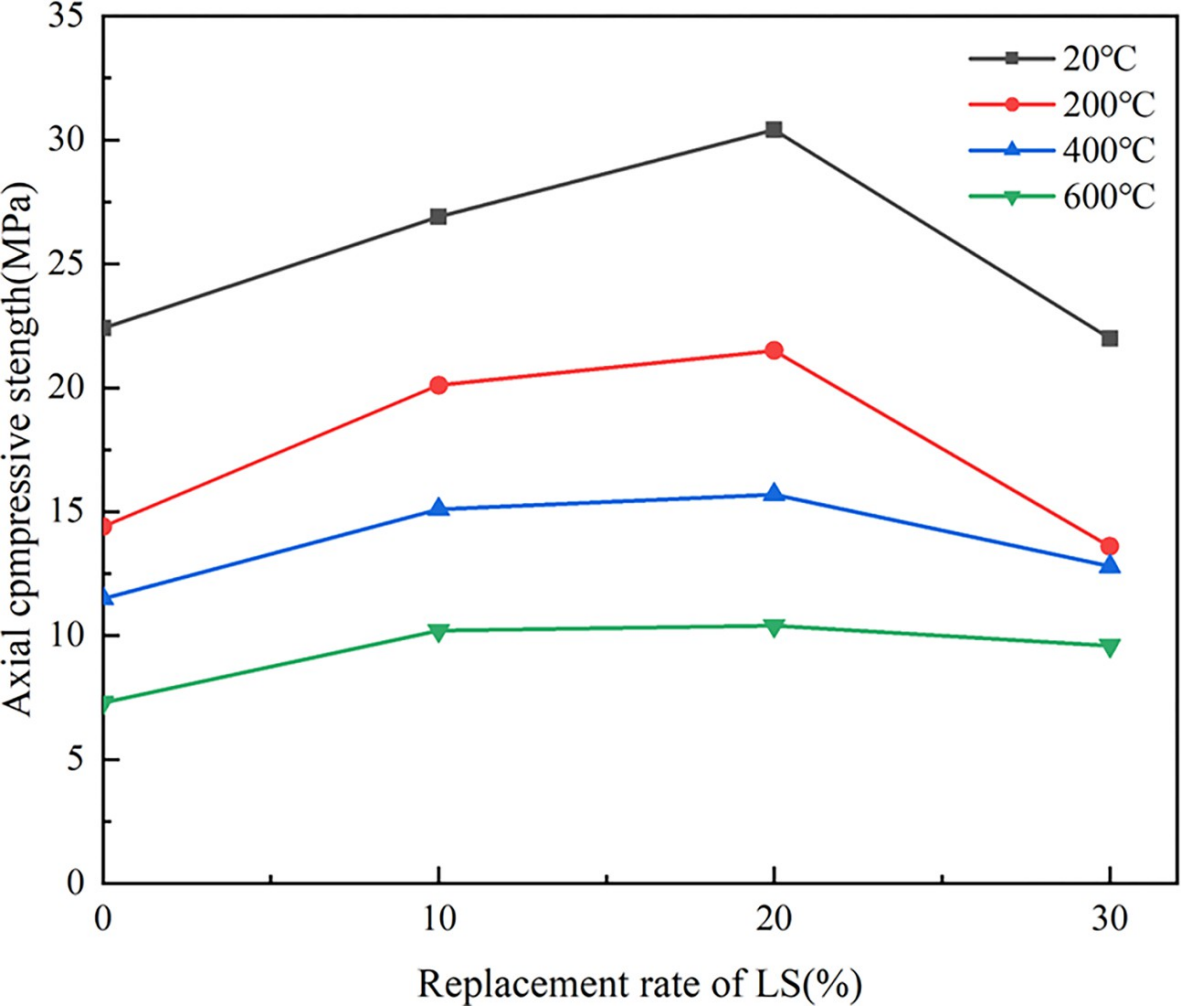
Axial compressive strength versus replacement of LS.

Fig 9 showed the axial compressive strength (*f*_*c*_) of LSRAC prismatic specimens versus temperature. With increasing of the temperature, *f*_*c*_ of the RAC specimens and LSRAC specimens were gradually decreasing trend. The *f*_*c*_ of the specimen decreased the fastest when reached 200°C, and its compressive strength retention rate varied between 61.6% and 74.9%. At 400°C, the decrease of *f*_*c*_ slowed down, in which the compressive strength retention rate of LSRAC with 30% lithium slag lowered by 3.4%. The *f*_*c*_ retention after high temperature action varied between 51.3% and 58.4%. At 600°C, the *f*_*c*_ of the specimens decreased faster and the retention varied between 32.6% and 43.5%. The *f*_*c*_ retention rate of LSRAC specimens with different admixtures of lithium slag was greater than that of RAC. The above analysis indicated that the temperature had a strong impact on *f*_*c*_ of LSRAC, and the higher the temperature, the more severe the loss. Fig 10 showed the *f*_*c*_ of LSRAC prismatic specimens versus the replacement rate of LS. With the 20% LS replacement rate, the axial compressive strength was optimized, which was increased by 36.5%-49.3% compared to RAC.

### 3.4 Splitting tensile strength

**Fig 11:**
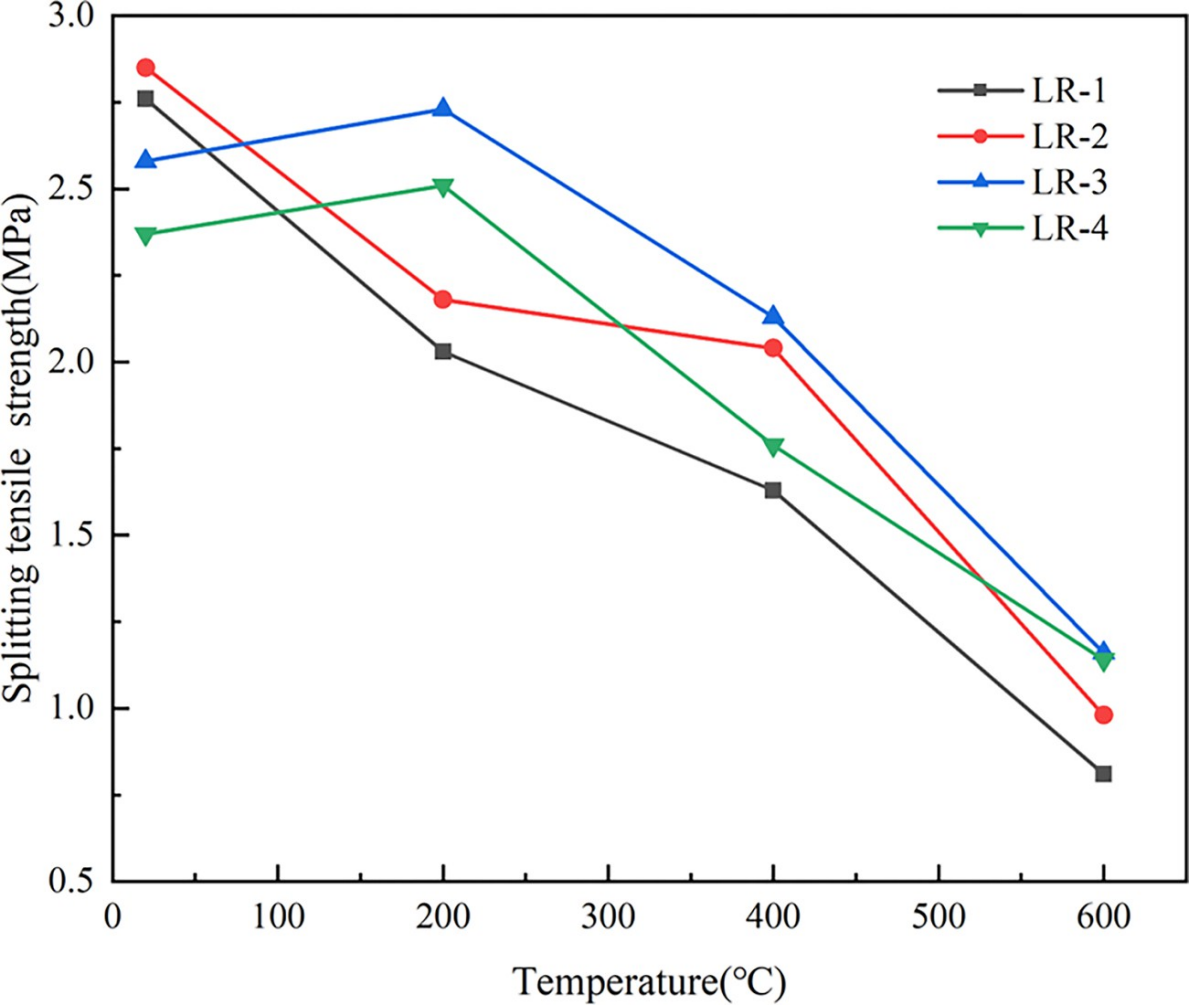
Splitting tensile strength versus temperature.

Fig 11 indicates that splitting tensile strength (*f*_*t*_) of LSRAC with 20% and 30% replacement rates increased first and then decreased with the temperature, while *ft* of other concrete gradually decreased. At 200°C, the retention of *ft* of RAC with 0% and 10% LS was 73.6% and 76.5%, and with 20% and 30%, the retentions were both about 105.8%. When heated to 600°C, the retention of RAC was 29.3%, and the retention of LSRAC varied between 34.4–48.1%. Since the *ft* of concrete mainly depended on the homogeneity of the material and the bonding strength of the aggregate, and the adhesion with mortar of RCA was poor, RCA with mortar on the surface caused the effect [44]. As the rate of substitution increased, the rate of increase of *f*_*t*_ decreased. With the increase in lithium slag dosage, *f*_*t*_ of LSRAC after high temperature climbed up and then declined. The reason for the sharp decrease of *f*_*t*_ after reaching 600°C may be that the hydrated calcium silicate gel in LSRAC was decomposed by the high temperature, which led to a reduction in the contact area between the interior of the concrete, a decrease in the adhesion between the aggregate and the slurry, made the *f*_*t*_ of LSRCA much lower.

**Fig 12:**
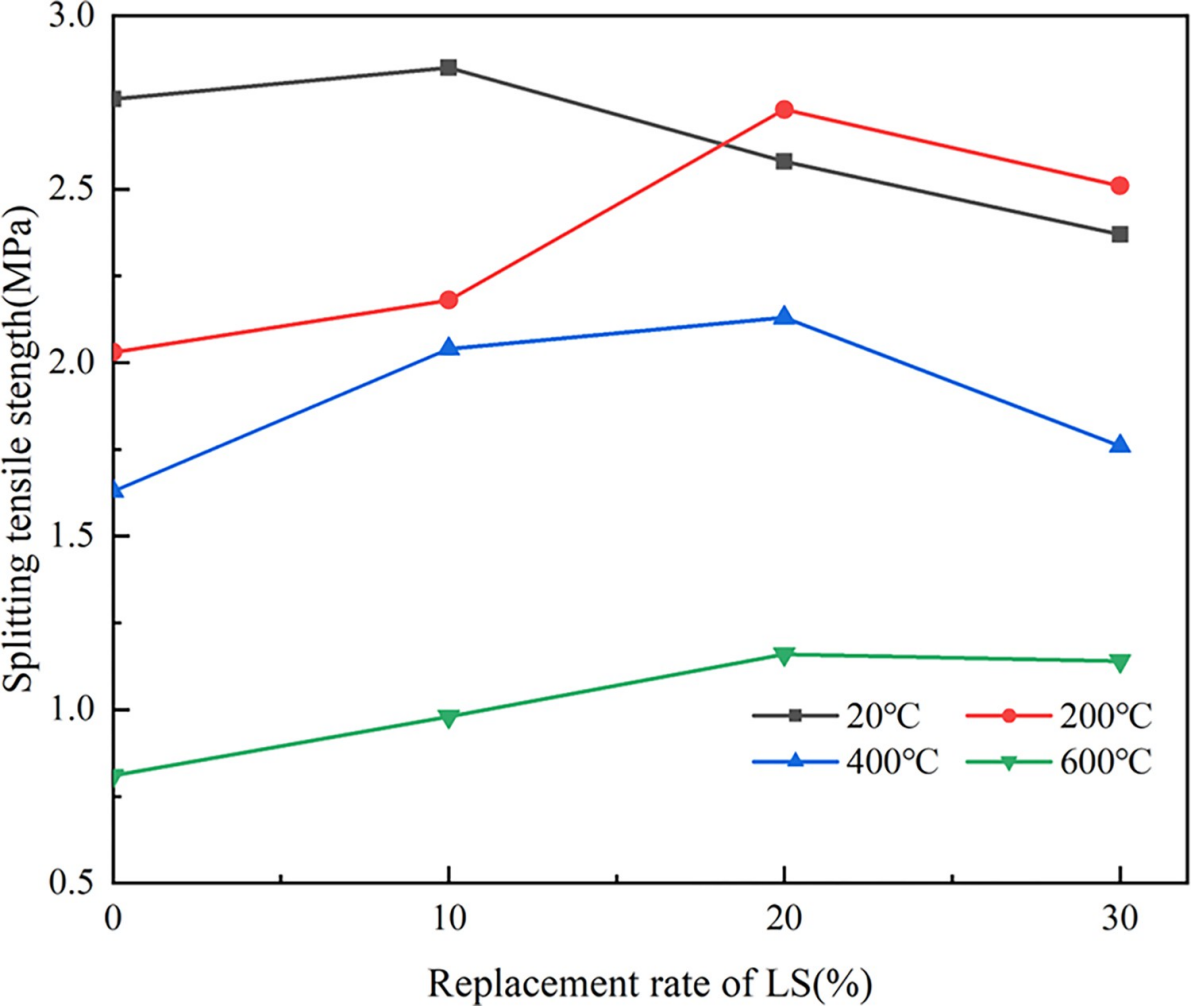
Splitting tensile strength versus replacement of LS.

As indicated in Fig 12, LS could enhance the interfacial structure between the aggregate and the cement, react with the cement and hydration products, and increase the secondary hydration of concrete and the bond of the corresponding interface [45]. *f*_*t*_ increased after mixing the appropriate amount of lithium slag. Excessive lithium slag was unable to fully involved in the secondary hydration reaction, and the rate of increase in *f*_*t*_ was reduced. Lithium slag could effectively improve the *f*_*t*_ of RAC specimens after elevated temperature, especially when the replacement rate was 20%, the *f*_*t*_ was optimal, increased by 30.7%-43.2%.

### 3.5 Flexural strength

At 20°C, the damage of RAC specimens was sudden, fractured into two halves after the peak load was reached, which was obvious brittle damage. LSRAC specimens without being heated weren’t observed cracks on the surface at the beginning of loading. With loading, some cracks began to appear at the bottom and gradually extended upward. When the load reached the maximum value, the specimen suddenly fractured. The brittleness of concrete increased with increasing temperature after being heated at high temperatures. As loading proceeds, the cracks due to high temperature action become wider. The cracks of the lower surface of the specimen were extended upward along the high temperature increase until the specimen split into two halves with a very irregular fracture surface.

**Fig 13:**
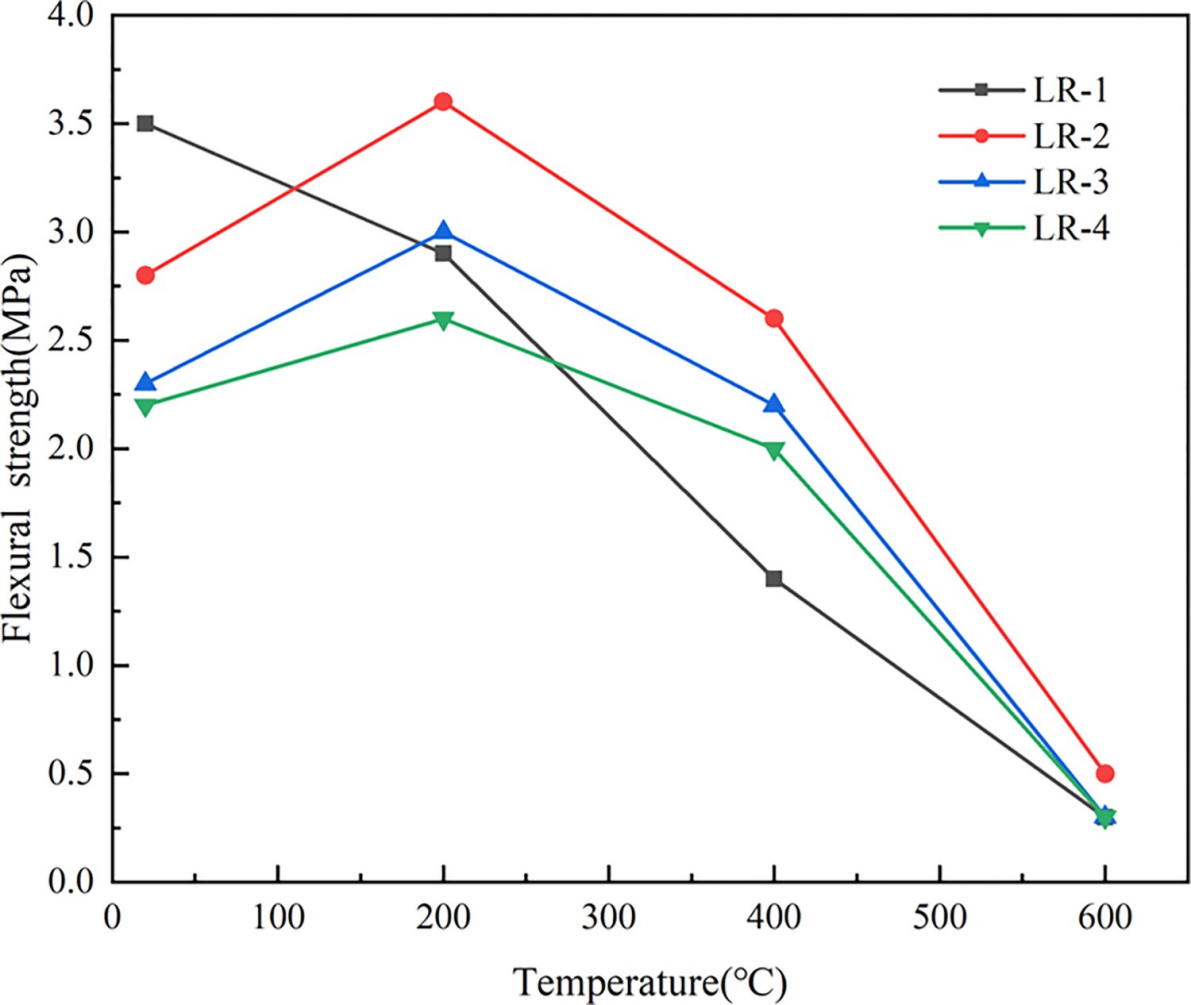
Flexural strength versus temperature.

Fig 13 indicated the trend of the flexural strength (*f*_*f*_) of LSRAC increasing first and then decreasing with the temperature. When the temperature was 200°C, the strength reached the maximum value, which was increased by 28.6%, 30.4%, and 18.2% for 10%, 20%, and 30% replacement rates, respectively. When 600°C, the strength dropped sharply. This was due to the presence of huge amounts of micro-cracks and micro-pores on the surface of RCA and mortar blocks covered its surface. These defects led to the existence of high water absorption, high porosity, high crushing index and low packing density of RCA, which affect the basic features of RAC [46]. On the one hand, after elevated-temperature calcination of LS, the hydration activity was improved, so that the strength of lithium slag concrete increased with the temperature [47, 48]; on the other hand, due to the mixing of RCA made the strength reduced, and finally t *f*_*f*_ reached the optimum at 200°C. When the temperature exceeds 400°C, the main reason for the decrease of *f*_*f*_ is the complete decomposition of the cement mortar. With increasing the temperature, the damaged parent aggregate began to decompose [35].

**Fig 14:**
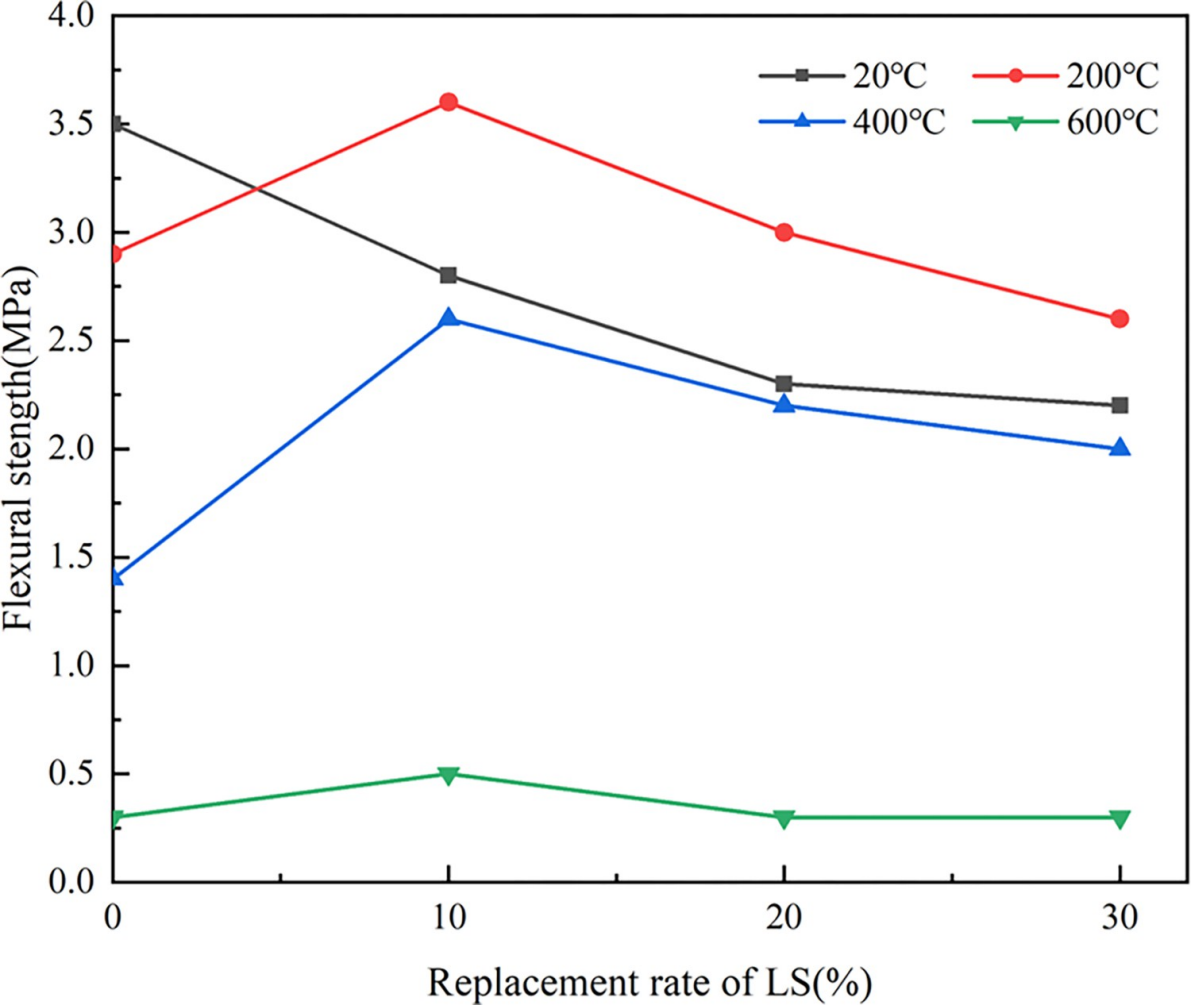
Flexural strength versus replacement of LS.

As shown in Fig 14, at 20°C, *f*_*f*_ of LSRAC declined with the growth of lithium slag admixture. However, after being heated, *f*_*f*_ of LSRAC specimens presented a trend of increasing firstly and then decreasing with the dosage of LS. When 10% dosage of LS, the greatest retention of *f*_*f*_ was 124.1%. After heating at high temperature, 10% was the optimum lithium slag replacement rate for LSRAC.

## 4. Strength retention equation

Based on analysis of the compressive strength of LSRAC after heating, it can be proposed that the relationship of compressive strength versus lithium slag dosage isn’t linear, as is the relationship with temperature. That is, with the lithium slag dosage or temperature increased, *f*_*c*_ presented a trend of increasing first and then decreasing or first decreasing and then increasing. In this paper, concerning to the formula presented by He et al. [45] for calculating *f*_*c*_ of heated plain concrete on the basis of the peak heating temperature, and taking into account the replacement ratio of LS, *f*_*c*_ formula of LSRAC after high temperature is proposed. The equation is as follows:

$$\frac{f_{c}^{T}}{f_{c}^{20}}=1.11757\times 10^{-6}\times T^{2}-0.00173\times T+1.01992\;20\leq T\leq 600$$ (1)

where T represents the heating temperature value of concrete, r represents the replacement rate value of lithium slag, *f*_*c*_^*20*^ denotes the compressive strength at 20°C, *f*_*c*_^*T*^ indicates the compressive strength at T°C.

The *f*_*t*_ and *f*_*f*_ have the same regularity as the compressive strength. The equations of the two strengths are as follows:

$$\frac{f_{t}^{T}}{f_{t}^{20}}=-1.43154\times 10^{-6}\times T^{2}-1.51293\times 10^{-4}\times T+1.00051\;20\leq T\leq 600$$ (2)

$$\frac{f_{f}^{T}}{f_{f}^{20}}=-5.30169\times 10^{-6}\times T^{2}+0.00175\times T+0.97889\;20\leq T\leq 600$$ (3)

**Fig 15:**
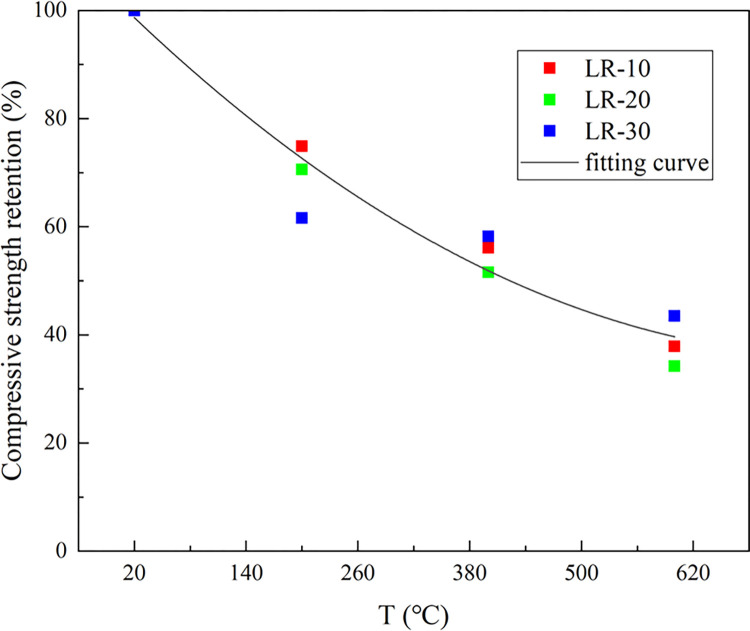
Fitting curve of compressive strength versus heating temperature.

**Fig 16:**
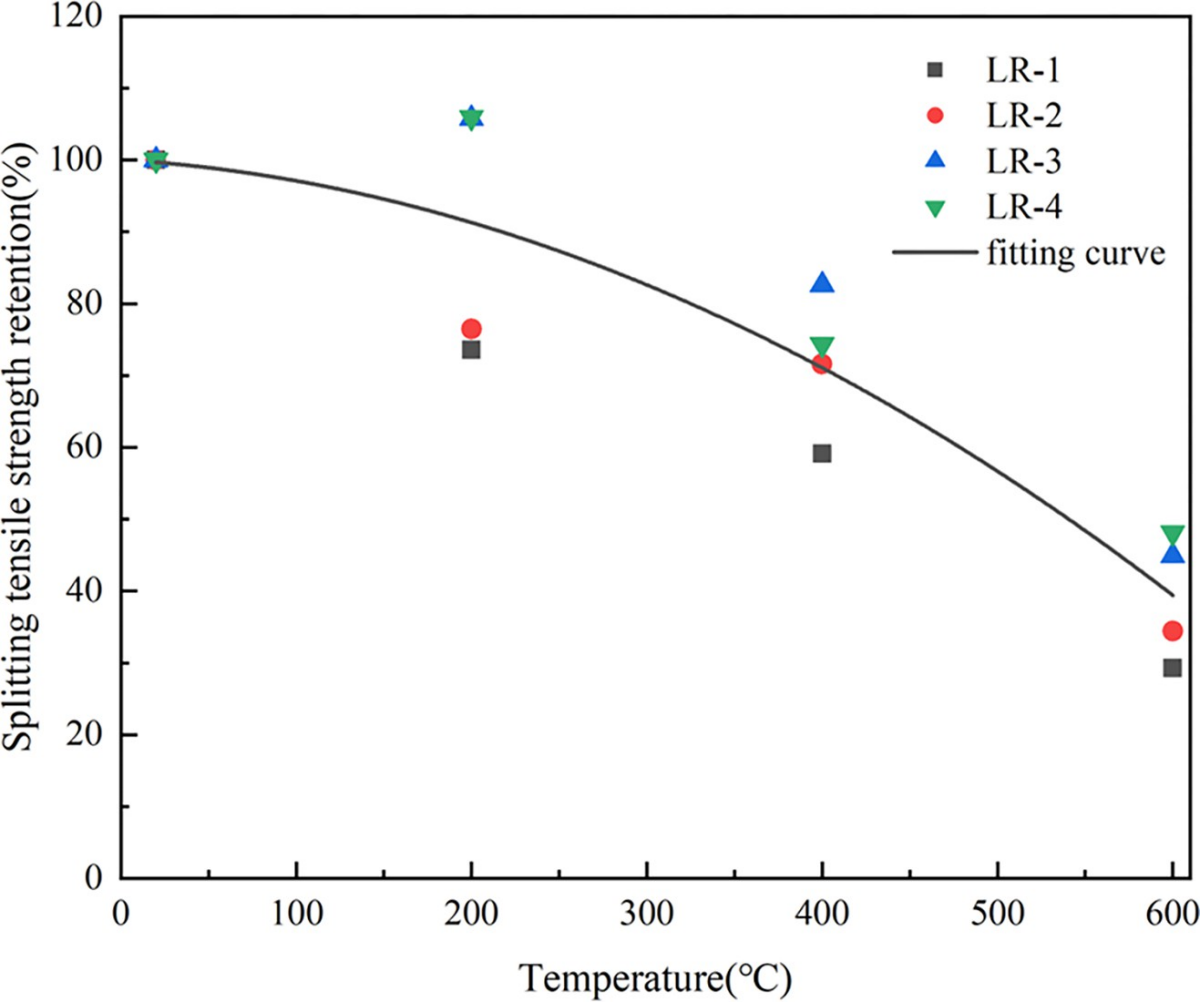
Fitting curve of splitting tensile strength versus heating temperature.

**Fig 17:**
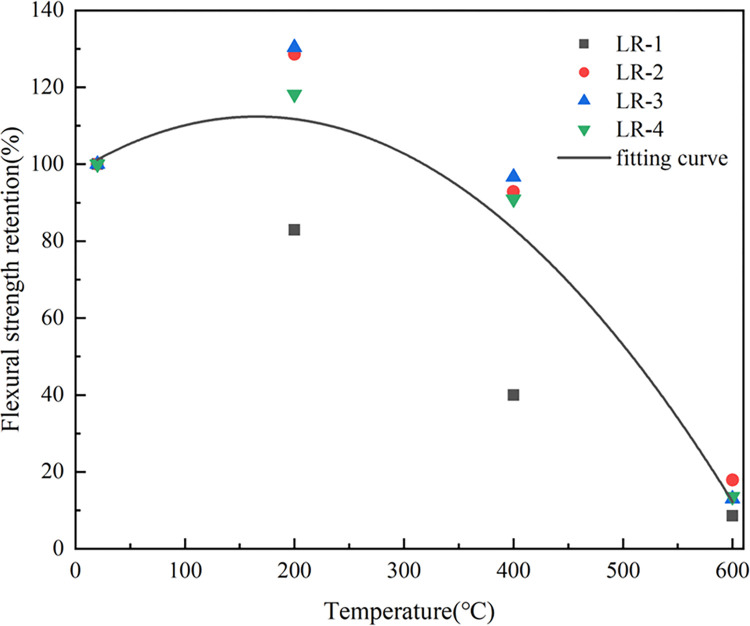
Fitting curve of flexural strength versus heating temperature.

**Fig 18:**
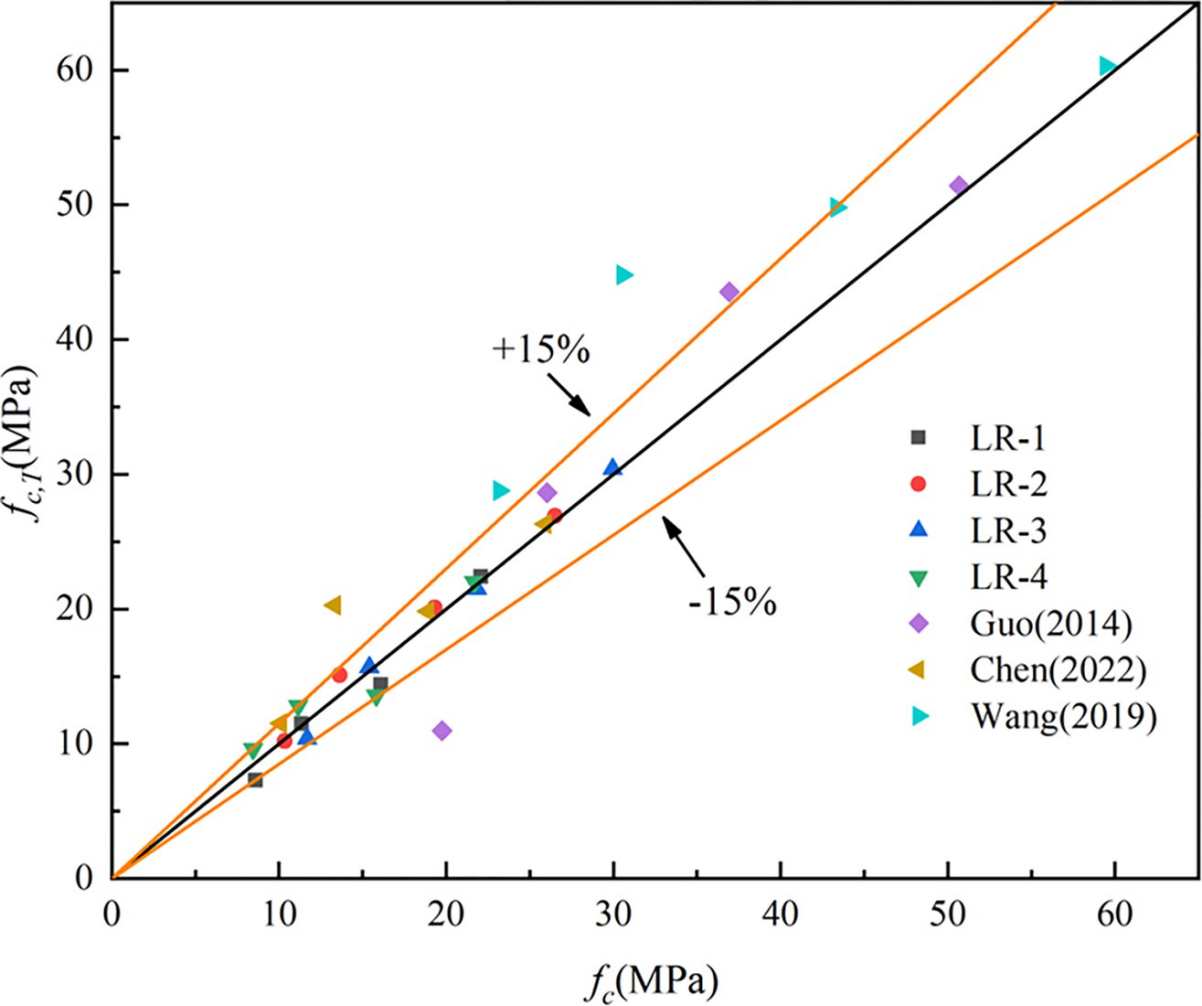
The predicted and test results for compressive strength.

**Fig 19:**
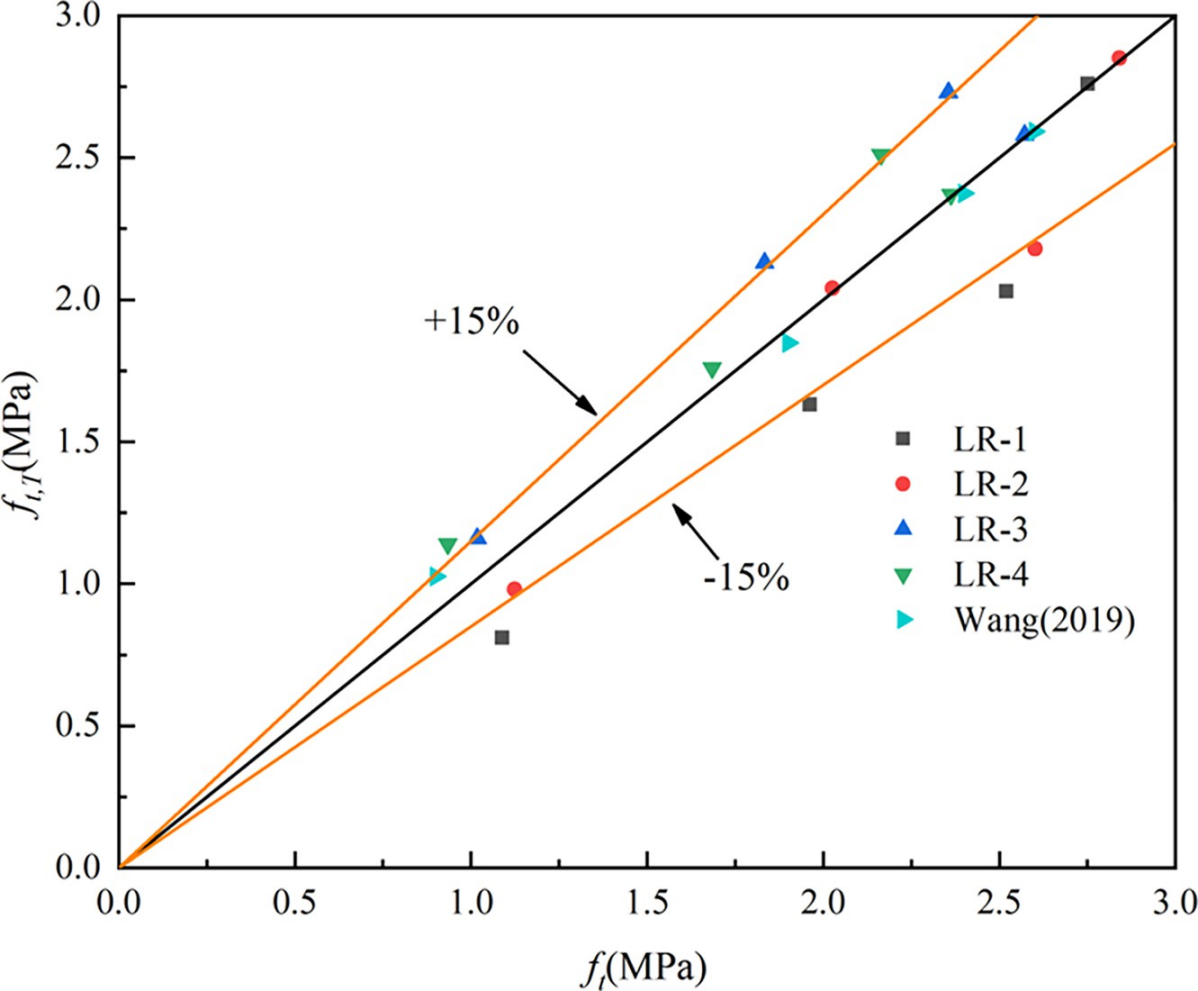
The predicted and test results for splitting tensile strength.

**Fig 20:**
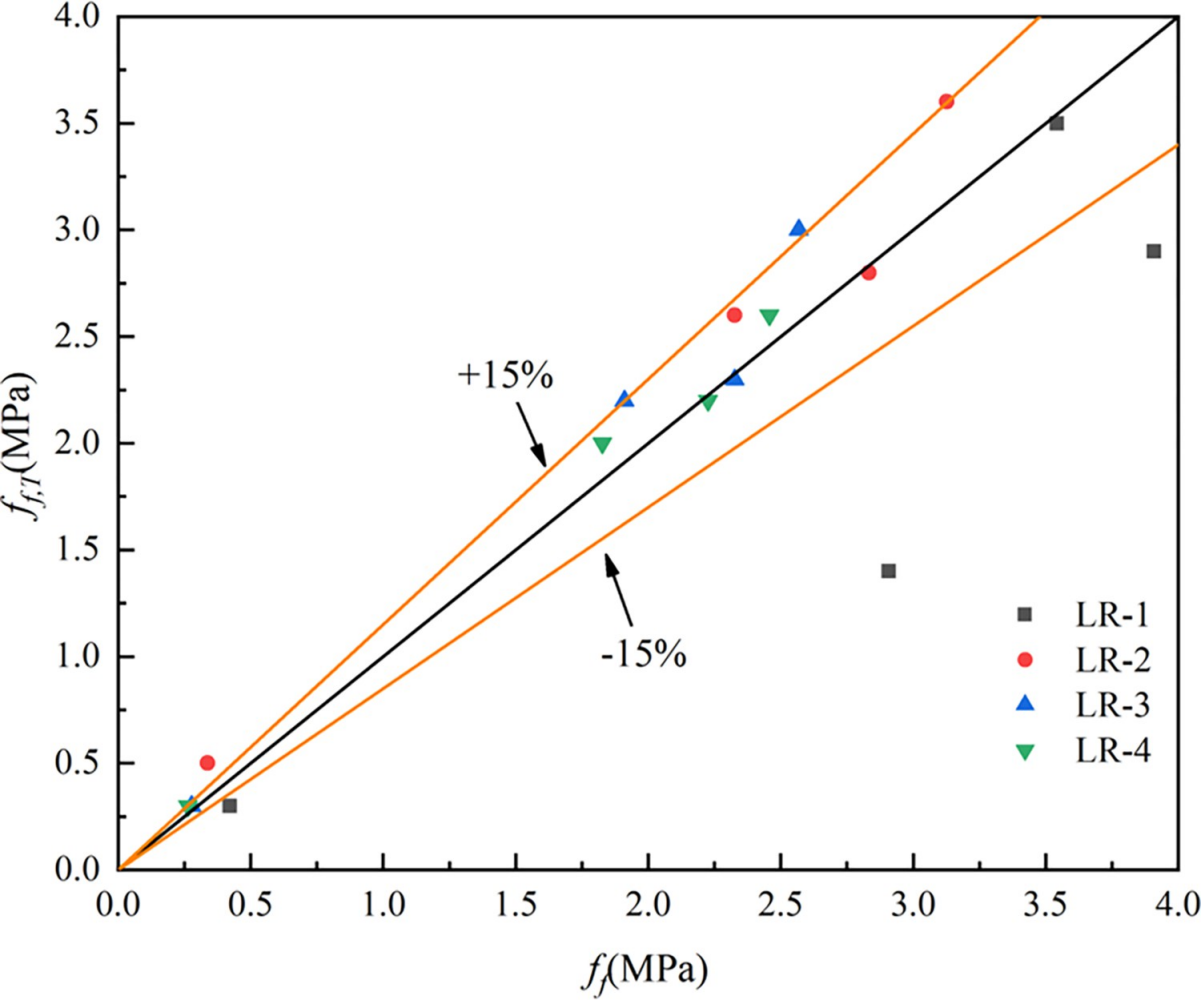
The predicted and test results for flexural strength.

Where *f*_*t*_^*20*^ indicates the splitting tensile strength at 20°C, *f*_*t*_^*T*^ represents the splitting tensile strength at T°C, *f*_*f*_^*20*^ denotes the flexural strength at 20°C and *f*_*f*_^*T*^ denotes the flexural strength at T°C. The R^2^ (coefficient of determination) of Eq (1), Eq (2) and Eq (3) were 0.96, 0.83 and 0.86. Figs 15–17 show the equations correlated with the test data well. Figs 18–20 displayed that most of the test data are within the 15% range, with the compressive and flexural strengths matching the references. Fig 18 does not validate other experimental results as no suitable referential data is found.

## 5. Conclusion

To improve the mechanical properties of RAC subject to high temperature, this work partially replaced cement with LS to make LSRAC. Meanwhile, this solved the problems associated with lithium slag pollution to the environment and reduced carbon emissions from cement production. This research used experimental data to analyze the effects of the replacement rate of LS and the heating temperature on the strengths of LSRAC. The conclusions were drawn as follows:

1. As the temperature rises, the surface change of aggregate is the same as that of ordinary concrete.
2. The high temperature greatly deteriorated the strengths of the concrete. If moderate amount of LS is added to the concrete, it can effectively alleviate the effect of high temperature on the surface deterioration of RAC.
3. After heating, the *f*_*cu*_, *f*_*c*_, and *f*_*t*_ of LSRAC with 20% of lithium slag admixture are optimal. Compared with RAC, the strengths are increased by 33.9%, 36.5%, and 34.5%, respectively. The *f*_*f*_ of LSRAC with 10% is optimal, increased by 24.1%. LS greatly improves the strengths of RAC compared to other supplementary cementitious materials [27] and can increase the bearing capacity if used in structural concrete.
4. The equations of the *f*_*c*_ retention, *f*_*t*_ retention, and *f*_*f*_ retention of LSRAC with different lithium slag substitution rates are proposed for the heating temperature, which the predicted results consist with the reference data. The formula offers theoretical guidance for on-site rescue, post-disaster assessment and reinforcement of RAC. Due to the lack of data, the relationship between quality and strengths can’t be well analyzed. It is recommended that follow-up research can increase the mixture ratio.
5. In this work, only the mechanical properties of LSRAC have been investigated, and its durability as well as components have to be further investigated in order to use LS extensively.
6. LS, as an industrial waste, can partially replace cement and improve the performance of RAC. If expanded the use is expanded, not only environmentally friendly treatment of lithium slag, but also to achieve cost reduction and improve the effectiveness of the engineering.

## Supporting information

S1 Data (XLSX)
